# Persistent Hypercalcemia Despite Parathyroidectomy for Primary Hyperparathyroidism in an Adult with Nephrocalcinosis and Nephrolithiasis Caused by a Novel Combination of Two Pathogenic *CYP24A1* Mutations

**DOI:** 10.3390/ijms26188965

**Published:** 2025-09-15

**Authors:** Sijun Zhang, Axel Muendlein, Edgar Meusburger, Emanuel Zitt

**Affiliations:** 1 Department of Internal Medicine 3 (Nephrology, Dialysis and Hypertension), LKH Feldkirch, 6800 Feldkirch, Austria; sijun.zhang@lkhf.at (S.Z.); edgar.meusburger@lkhf.at (E.M.); 2 Vorarlberg Institute for Vascular Investigation and Treatment (VIVIT), 6800 Feldkirch, Austria; axel.muendlein@vivit.at; 3 Agency for Preventive and Social Medicine (aks), 6900 Bregenz, Austria

**Keywords:** *CYP24A1* mutation, hypercalcemia, hyperparathyroidism, nephrocalcinosis, vitamin D

## Abstract

Hypercalcemia is a frequent electrolyte disorder with a wide range of possible causes. While primary hyperparathyroidism is one of the most frequent causes, loss-of-function mutations in the *CYP24A1* gene, which encodes for the 24-hydroxylase enzyme responsible for the catabolism of 25(OH)D_3_ and 1,25(OH)_2_D_3_, have been described as a rare cause of hypercalcemia associated with nephrocalcinosis and nephrolithiasis due to the reduced degradation of vitamin D metabolites. We describe an interesting case of a 67-year-old woman who suffered from hypercalcemia with nephrocalcinosis and nephrolithiasis caused by the simultaneous presence of these two conditions. At the first presentation, primary hyperparathyroidism due to parathyroid adenoma was found to be causative, with partial parathyroidectomy leading to temporary normocalcemia. As hypercalcemia reappeared, an elevated 25(OH)D_3_/24,25(OH)_2_D3 ratio and consequently a novel combination of two pathogenic heterozygous missense mutations (c.1186C>T and c.628T>C) of the *CYP24A1* gene were found. This case highlights the diagnostic complexity of persistent hypercalcemia and underscores the importance of also considering rare causes such as *CYP24A1* mutations in the differential diagnosis after the exclusion of relevant frequent disease causes.

## 1. Introduction

Hypercalcemia is one of the most frequently encountered electrolyte disorders in clinical practice [1]. From a diagnostic perspective, the causes of hypercalcemia can be divided into the following categories: PTH-dependent and PTH-independent. Elevated PTH levels accompanying hypercalcemia indicate primary hyperparathyroidism (pHPT) or, less frequently, tertiary hyperparathyroidism in patients with long-standing severe chronic kidney disease, or persistent post-transplant hyperparathyroidism after successful kidney transplantation in patients with secondary hyperparathyroidism prior to transplantation. Low-normal or suppressed PTH narrows down the PTH-independent causes, which can be roughly divided into the three most frequent groups of (i) malignancy-associated, (ii) drug-related, or (iii) vitamin D-mediated hypercalcemia. The latter group encompasses hypervitaminosis D due to the administration of excessive amounts of vitamin D or 25(OH)D_3_, granulomatous or lymphoproliferative diseases with excessive endogenous production of 1,25(OH)_2_D_3_, and rarely mutations in enzymes resulting in reduced degradation of 25(OH)D_3_ and 1,25(OH)_2_D_3_ [2]. Loss-of-function mutations in the *CYP24A1* gene, which encodes for the 24-hydroxylase enzyme responsible for the catabolism of 25(OH)D_3_ and 1,25(OH)_2_D_3_, have been described as a rare cause of hypercalcemia associated with nephrocalcinosis and nephrolithiasis, since its first description in 2011 in patients with so-called idiopathic infantile hypercalcemia [3,4]. Earlier, our group found a homozygous new pathogenic missense mutation (c.628T>C) in exon 4 of the gene, causing tryptophan to be replaced with arginine in codon 210 [5]. Here, we describe an interesting case of a 67-year-old woman who suffered from hypercalcemia with nephrocalcinosis and nephrolithiasis caused by a novel finding of the simultaneous presence of pHPT as a frequent cause, and a novel combination of two pathogenic *CYP24A1* mutations as a very rare cause of hypercalcemia.

## 2. Case Report

**Figure 1:**
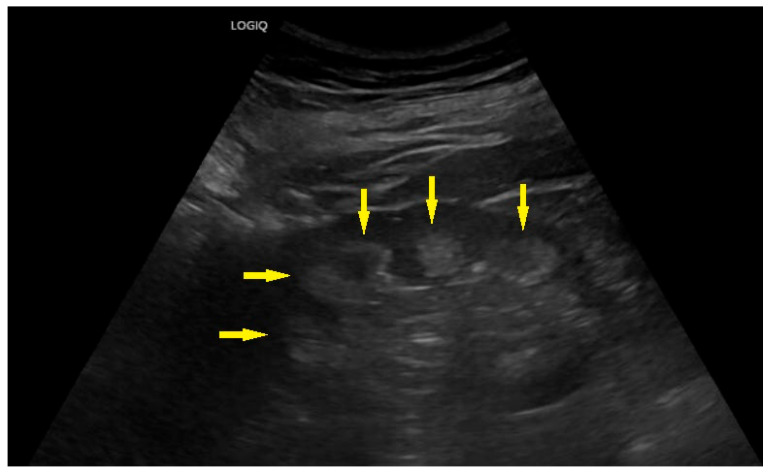
Ultrasound of the right kidney showing marked medullary nephrocalcinosis (marked by arrows).

A 67-year-old female patient was referred to our nephrology department from her general practitioner in 2023 for the evaluation of chronic kidney disease (CKD). Her CKD stage at that time was G3aA1. The most striking finding was the renal ultrasound, which showed typical features of medullary nephrocalcinosis (Figure 1).

Her medical history was notable for recurrent episodes of urolithiasis, requiring multiple urological interventions. Primary hyperparathyroidism was suspected in the past as the underlying cause, given her inadequately high-normal parathyroid hormone levels (57 pg/mL, October 2018) despite hypercalcemia (total calcium 3.22 mmol/L). Sonographic evaluation revealed a parathyroid adenoma, which led to a partial parathyroidectomy in February 2019. Postoperatively, her PTH levels dropped significantly (8 pg/mL).

Another interesting fact was a history of frequent abdominal pain, which led to repeated gastroscopy, revealing varying degrees of gastritis. In 2017, a Helicobacter pylori-associated MALT lymphoma was histologically diagnosed. After eradication treatment, the lymphoma remitted. Follow-up gastroscopies in 2018, 2020, and 2023 showed maintained remission.

At the time of a nephrological evaluation in June 2023, hypercalcemia had reappeared. Total serum calcium was 2.75 mmol/L, and ionized calcium was 1.37 mmol/L. An initial workup for a recurrence of pHPT was conducted. However, repeat sonography and SPECT imaging showed no parathyroidal abnormalities, leading to further differential diagnostic assessments.

Other common causes of hypercalcemia were excluded as follows: There was no indication of a myeloma disorder, as the patient had a negative serum and urine immunofixation, unremarkable serum protein electrophoresis, and a normal serum free light chain ratio and urinary free light chain excretion. Parathyroid hormone-related protein (PTHrP) was undetectable (<1 pmol/L), making a malignancy-related cause of hypercalcemia unlikely. Her long-term medication did not include any typical drugs that could cause hypercalcemia. 25-hydroxyvitamin D and 1,25-dihydroxyvitamin D levels were in the upper normal range (25(OH)D3: 51 µg/L, [reference range: 30–70 µg/L], 1,25(OH)_2_D3: 60.4 ng/L, [reference range: 19–79 ng/L]). Clinically, there were no signs of a granulomatous disease.

The 1,25(OH)_2_D_3_ value was considered inadequately elevated because hypercalcemia was present at that time, which should physiologically suppress alpha-1-hydroxylase (CYP27B1) activity. Disorders in the metabolic breakdown of vitamin D have been described as rare causes of hypercalcemia. Therefore, the vitamin D catabolism was investigated through the determination of its breakdown products 25(OH)D_3_, 25(OH)D_2_, 24,25(OH)_2_D_3_, and 3c-epimer 25(OH)D_3_ by liquid chromatography with tandem mass spectrometry (LS-MS/MS, MVZ Labor Dr. Limbach, Heidelberg, Germany). This revealed a low 24,25(OH)_2_D_3_ concentration (1.3 µg/L) and an elevated 25(OH)D_3_/24,25(OH)_2_D_3_ ratio of 39.2, supporting our hypothesis of a causative disorder in the vitamin D metabolism.

Vitamin D 24-hydroxylase (CYP24A1) is an important enzyme in the regulation of vitamin D. To date, many different *CYP24A1* mutations have already been described in the literature as causes of vitamin D metabolism disorders. Sequence analysis of the *CYP24A1* gene was performed in our patient to confirm the diagnosis. For this purpose, all exons, including the intron–exon boundaries of the *CYP24A1* gene, were amplified by polymerase chain reaction (PCR) according to a previously published protocol by Tebben et al. [6]. The PCR products were subjected to direct sequencing at an external sequencing facility (Microsynth AG, 9436 Balgach, Switzerland).

**Figure 2:**
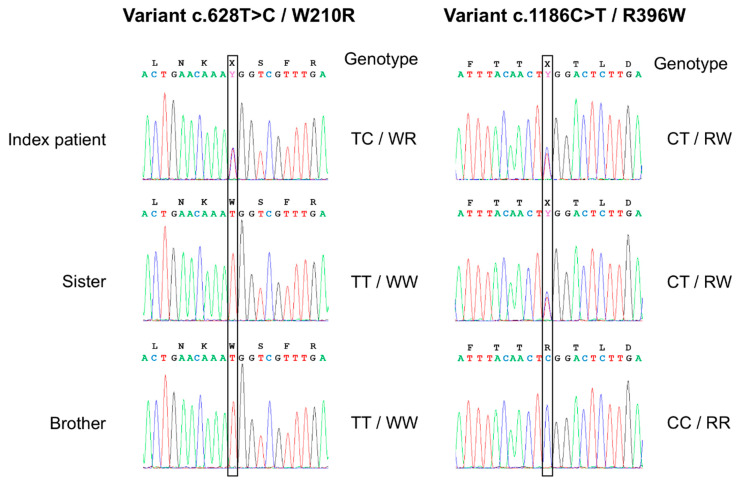
Genotyping for the c.628T>C/W210R and c.1186C>T/R396W mutation by DNA sequence analysis in the index patient and her siblings. DNA sequencing chromatographs are shown for both genotypes and illustrate the transversion of T to C, which causes an amino acid change from tryptophan (W) to arginine (R) in codon 210, and the transversion of C to T resulting in an amino acid change from arginine (R) to tryptophan (W) in codon 396, respectively.

Sequencing analysis revealed that the patient was heterozygous for three common DNA polymorphisms (rs2296241, rs2762934, and rs10623012) and additionally for two sequence variants of particular significance. According to HGVS nomenclature, based on the MANE Select transcript of *CYP24A1* (NM_000782.5; protein: NP_000773.2), these variants are designated as NM_000782.5:c.1186C>T, NP_000773.2:p.Arg396Trp (R396W), rs114368325 and NM_000782.5:c.628T>C, NP_000773.2:p.Trp210Arg (W210R) (Figure 2). In silico analysis of the W210R and R396W mutation using the SIFT tool [7] (http://sift.jcvi.org, accessed on 15 December 2023) and the PolyPhen-2 tool [8] (http://genetics.bwh.harvard.edu/pph2/index.shtml, accessed on 15 December 2023) consistently showed a probably damaging effect on the protein, reaching the highest possible scores of the two algorithms (SIFT score: 0.00; PolyPhen-2 score: 1.000).

Both of them have been described with “infantile hypercalcemia-1” (HCINF1, OMIM #143880), the c.628T>C variant identified by our group in an earlier independent patient in 2013 [5], and are classified as pathogenic according to the ACMG/AMP 2015 guidelines [9]. The co-occurrence of these two heterozygous variants in our patient suggests compound heterozygosity. This assumption is further supported by the segregation analysis, as the patient’s sister was found to carry only the c.1186C>T (R396W) variant in the heterozygous state, while the brother carried neither variant. Therefore, this constellation makes it likely that the two variants are located on different alleles (in trans), consistent with a compound heterozygous state. The c.628T>C variant, first described in another patient within our nephrology department, has not been reported again in the literature since then, whereas the c.1186C>T variant in the homozygous or compound heterozygous state has been reported in almost 50 patients so far, as summarized in a comprehensive review including 221 patients with detailed genetic analyses [3]. A familial relationship between the two affected patients carrying the c.628T>C variant seems likely. However, we were unable to establish this based on a detailed family history. Both siblings of the index patient presented with normal calcium and PTH concentrations, and an unremarkable 25(OH)D_3_/24,25(OH)_2_D_3_ ratio (Table 1). These findings are consistent with an autosomal recessive mode of inheritance. The mildly decreased kidney function (eGFR 55 mL/min/1.73 m^2^) in the index patient’s sister was due to vascular kidney disease, as she suffered from arterial hypertension, hyperlipidemia, coronary artery disease, and moderate aortic valve stenosis.

Therapeutically, our patient was advised to avoid any vitamin D or calcium supplementation, to prevent excessive sunlight exposure, and to use sufficient sun protection. Two years after diagnosis, she remained mildly hypercalcemic (total serum calcium 2.66 mmol/L, ionized calcium 1.38 mmol/L), with PTH levels in the low-normal range (20 pg/mL) and stable kidney function (eGFR 55 mL/min/1.73 m^2^).

## 3. Discussion

This case highlights the diagnostic complexity of persistent hypercalcemia and underscores the importance of also considering rare causes such as *CYP24A1* mutations in the differential diagnosis, after the exclusion of relevant frequent disease causes.

Our index patient initially presented with a classical picture of pHPT, including hypercalcemia, an inappropriately normal PTH concentration, and a sonographically and histologically confirmed parathyroid adenoma. Surgical removal led to the expected PTH reduction and transient normocalcemia. However, recurrence of hypercalcemia with suppressed PTH was incompatible with the original diagnosis and required further investigation. Subsequent laboratory findings revealed an abnormally high 25(OH)D_3_/24,25(OH)_2_D_3_ ratio, indicating impaired 24-hydroxylation in vitamin D metabolism [10,11]. Genetic analysis confirmed two mutations in the *CYP24A1* gene (W210R and R396W) in a compound heterozygous state, both of which have been described as pathogenic [3].

The co-occurrence of a pathogenic *CYP24A1* gene mutation and pHPT is rare. Only a few cases have been reported so far [12,13,14,15,16,17]. It remains unclear whether the combination of pHPT and *CYP24A1* mutation is just a coincidence or causally linked. To date, no case of a familial pHPT with a chromosomal aberration or mutation on chromosome 20q13, where the *CYP24A1* gene is encoded, has been described. Mutations in the *CDC73*, *GCM2*, *CaSR*, and *CCND1* genes have been found in patients with isolated familial pHPT. Furthermore, pHPT could also be the leading phenotype in patients with variable expression of multiple endocrine neoplasia due to a *MEN1* mutation [18]. At first glance, both causes of hypercalcemia differ clearly in concomitant PTH concentrations. While pHPT is characterized by elevated or inappropriately normal PTH values, PTH levels are usually suppressed in patients with 24-hydroxylase deficiency due to *CYP24A1* mutations. Our patient had a PTH in the lower third of the normal range, probably caused by the mildly decreased kidney function. Interestingly, the patient’s sister, who was heterozygous for the c.1186C>T (R396W) mutation, presented with a low 24,25(OH)_2_D_3_ concentration (0.61 µg/L), a value even lower than that found in our index patient. This low concentration is most likely caused by the low 25(OH)D_3_ concentration (12 µg/L), because with a low substrate concentration (25(OH)D_3_), the degradation product will also decrease. In contrast, the 25(OH)D_3_/24,25(OH)_2_D_3_ ratio will not change significantly [2]. Consistent with this, her ratio was still in the reference range, as most heterozygotes and unaffected patients have a ratio of <30 [2].

The detected sequence variants are exceedingly rare. Carriers of the c.628T>C variant have been reported in only the present report and in the work by Meusburger et al. [5]. The mutations follow an autosomal recessive inheritance pattern, in which heterozygous carriers remain unaffected, while disease manifestation requires biallelic variants, either in the homozygous or compound heterozygous states.

Primary hyperparathyroidism and genetic 24-hydroxylation defects differ in treatment modalities. While pHPT is surgically curable, *CYP24A1* mutation-associated hypercalcemia requires conservative management. This includes the strict avoidance of vitamin D and calcium supplements, a calcium-restricted diet, reduced UV exposure, and the pharmacological inhibition of the 1α-hydroxylase using azole derivatives [2,5,19]. Whereas CYP24A1 induction with glucocorticoids does not sufficiently work in patients with genetically reduced CYP24A1 activity, fluconazole [20] and ketoconazole [21] have both been shown to reduce 1,25(OH)_2_D_3_ and normalize hypercalcemia in CYP24A1-deficient patients. Ketoconazole inhibits the hydroxylation activity of CYP27B1 which has been shown in vitro in human renal tubular cell lines [22]. Another pharmaceutical therapeutic option is the enzymatic induction of CYP3A4 by rifampicin, which has been successfully used in patients with *CYP24A1* mutations to diminish hypercalcemia and hypercalciuria [23]. Like CYP24A1, hepatic CYP3A4 inactivates 25(OH)D_3_ and 1,25(OH)_2_D_3_. This degradation step is not regulated by PTH or vitamin D metabolites, but can be significantly induced by certain drugs such as rifampicin [24]. However, relevant side effects will prevent the long-term use for both modalities. As a limitation of our study, a further characterization of the phenotype, including an extended family history of the index patient, was not possible because the index patient was childless, and both of her parents had already passed away.

## 4. Conclusions

This case illustrates that even if an obvious cause for hypercalcemia with nephrocalcinosis and nephrolithiasis, such as pHPT, is present, rare causes such as genetic mutations should never be forgotten. Adapting the Woodward aphorism, in rare cases, it is not only the horses but also a zebra that contributes to the clatter of hooves. In a first step, PTH-dependent and -independent causes of hypercalcemia can be easily separated. In the latter, within the group of vitamin D-mediated causes, an increased 25(OH)D_3_/24,25(OH)_2_D_3_ ratio gives the first hint of an impaired enzymatic vitamin D metabolism, which could be caused by loss-of-function mutations in the *CYP24A1* gene, as in our case.

## Figures and Tables

**Table 1:** Laboratory parameters of the index patient and siblings with mutational status.

Family Member	Patient	Sister	Brother	Reference Range
**Age (years)**	68	79	75	
**Gender**	female	female	male	
**Nephrolithiasis**	yes	no	no	
**Nephrocalcinosis**	yes	no	no	
**c.628T>C/W210R, genotype**	heterozygous, TC/WR	wildtype, TT/WW	wildtype, TT/WW	
**c.1186C>T/R396W, genotype**	heterozygous, CT/RW	heterozygous, CT/RW	wildtype, CC/RR	
**Total Calcium (mmol/L)**	2.75	2.37	2.31	2.15–2.55
**Ionized Calcium (mmol/L)**	1.37	1.19	1.17	1.12–1.32
**Phosphate (mmol/L)**	1.24	1.09	1.01	0.81–1.45
**PTH (pg/mL)**	27	64	40	15–65
**25(OH)D_3_ (µg/L)**	51	12	20	20–70
**1,25(OH)_2_D_3_ (ng/L)**	60.4	uk	uk	19.9–79.3
**24,25(OH)_2_D_3_ (µg/L)**	1.3	0.61	2.5	1–3
**25(OH)D_3_/24,25(OH)_2_D_3_**	39.2	19.7	8	7–35
**Creatinine (mg/dL)**	1.05	0.98	0.64	0.50–0.90 (f), 0.70–1.20 (m)
**eGFR (mL/min/1.73 m^2^)**	55	55	96	>60
**FGF-23 c-terminal (pg/mL)**	192	uk	uk	23.2–95.4

Abbreviations: PTH: parathyroid hormone; eGFR, estimated CKD-EPI creatinine-based glomerular filtration rate; FGF-23, fibroblast growth factor 23; uk, unknown.

## Data Availability

The original contributions presented in the study are included in the article. Further inquiries can be directed to the corresponding author.
